# Long-term prognosis of chronic total occlusion treated by successful percutaneous coronary intervention in patients with or without diabetes mellitus: a systematic review and meta-analysis

**DOI:** 10.1186/s12933-021-01223-8

**Published:** 2021-01-30

**Authors:** Yong Zhu, Shuai Meng, Maolin Chen, Kesen Liu, Ruofei Jia, Hong Li, Huagang Zhu, Zening Jin

**Affiliations:** 1 Department of Cardiology, Beijing Anzhen Hospital, Capital Medical University, Beijing, 100029 China; 2 Department of Cardiology, Beijing Tiantan Hospital, Capital Medical University, Beijing, 100029 China

**Keywords:** Diabetes mellitus, Percutaneous coronary intervention, Medical treatment, Chronic total occlusions, Major adverse cardiac events

## Abstract

**Background:**

Diabetes mellitus (DM) is highly prevalent among patients undergoing percutaneous coronary intervention (PCI) for chronic total occlusion (CTO). Therefore, the purpose of our study was to investigate the clinical outcomes of CTO-PCI in patients with or without DM.

**Methods:**

All relevant articles published in electronic databases (PubMed, Embase, and the Cochrane Library) from inception to August 7, 2020 were identified with a comprehensive literature search. Additionally, we defined major adverse cardiac events (MACEs) as the primary endpoint and used risk ratios (RRs) with 95% confidence intervals (CIs) to express the pooled effects in this meta-analysis.

**Results:**

Eleven studies consisting of 4238 DM patients and 5609 non-DM patients were included in our meta-analysis. For DM patients, successful CTO-PCI was associated with a significantly lower risk of MACEs (RR = 0.67, 95% CI 0.55–0.82, *p* = 0.0001), all-cause death (RR = 0.46, 95% CI 0.38–0.56, *p* < 0.00001), and cardiac death (RR = 0.35, 95% CI 0.26–0.48, *p* < 0.00001) than CTO-medical treatment (MT) alone; however, this does not apply to non-DM patients. Subsequently, the subgroup analysis also obtained consistent conclusions. In addition, our study also revealed that non-DM patients may suffer less risk from MACEs (RR = 1.26, 95% CI 1.02–1.56, *p* = 0.03) than DM patients after successful CTO-PCI, especially in the subgroup with a follow-up period of less than 3 years (RR = 1.43, 95% CI 1.22–1.67, *p* < 0.0001).

**Conclusions:**

Compared with CTO-MT alone, successful CTO-PCI was found to be related to a better long-term prognosis in DM patients but not in non-DM patients. However, compared with non-DM patients, the risk of MACEs may be higher in DM patients after successful CTO-PCI in the drug-eluting stent era, especially during a follow-up period shorter than 3 years.

## Introduction

Coronary chronic total occlusion (CTO), which is defined as a native coronary artery that is completely obstructed with Thrombolysis In Myocardial Infarction (TIMI) grade 0 flow for more than 3 months, is observed in approximately 15–25% of patients undergoing diagnostic coronary angiography[1, 2]. Considerable evidence suggests that successful percutaneous coronary intervention (PCI) of CTO lesions is associated with a greater improvement of symptoms, quality of life, and left ventricular function compared with failed CTO-PCI or initial medical treatment (MT) alone [3–5]. However, the beneficial effect of CTO-PCI on long-term survival remains controversial [1, 6]. Therefore, current guidelines recommending CTO-PCI, such as the IIa B recommendation, should be considered in patients with severe angina resistant to MT or with a large ischaemic area in the territory of the occluded vessel [7].

Diabetes mellitus (DM), a well-known coronary artery disease (CAD) risk factor, is associated with a greater atherosclerotic burden, including diffuse CAD, multivessel disease, and heavy coronary artery calcifications [8, 9]. DM is also relatively common in coronary CTO patients (approximately 30–40%), which suggests that DM may be a risk factor for CTO [10, 11]. In addition, CTO patients with DM are more likely to have longer CTO lesions and higher Japanese-chronic total occlusion (J-CTO) scores [12]. The existence of DM could also have a detrimental effect on collateral circulation development and microcirculation function [5, 13–15]. Therefore, DM is an important risk factor, and we should take it into account when determining treatment regimens for stable patients with CTO.

Currently, the limited data regarding the clinical outcomes of CTO-PCI in patients with or without DM mostly rely on observational studies rather than randomized controlled trials (RCTs), and the results remain controversial. Therefore, it is necessary for us to perform a meta-analysis of eligible studies to investigate the long-term prognosis of successful CTO-PCI in patients with or without DM.

## Methods

We performed the present meta-analysis following the PRISMA (Preferred Reporting Item for Systematic Reviews and Meta-Analysis) statement [16]. Additionally, we also registered this trial in PROSPERO, number CRD42020201119.

### Search strategy

A comprehensive literature search was performed to identify all relevant articles published from inception to August 7, 2020 in the PubMed, Embase, and Cochrane Library databases. The search terms used in the present study are as follows: diabetes mellitus OR diabetes OR type 2 diabetes mellitus OR diabetes mellitus, type 2 OR T2DM OR type 1 diabetes mellitus OR diabetes mellitus, type 1 OR T1DM OR DM; chronic total occlusion OR chronic total coronary occlusion OR chronic total occlusions OR chronic total coronary occlusions OR CTO; percutaneous coronary intervention OR coronary intervention, percutaneous OR coronary interventions, percutaneous OR intervention, percutaneous coronary OR interventions, percutaneous coronary OR percutaneous coronary interventions OR percutaneous coronary revascularization OR coronary revascularization, percutaneous OR coronary revascularizations, percutaneous OR percutaneous coronary revascularizations OR revascularization, percutaneous coronary OR revascularization, percutaneous coronary OR percutaneous coronary angioplasty OR PCI. The reference lists of eligible studies were also checked manually to identify additional relevant articles. The details of our search strategy are presented in the supplementary material.

### Inclusion and exclusion criteria

The study selection was performed manually by two investigators (RFJ and SM) with the assistance of EndNote software. Studies that met the following criteria were included in the present meta-analysis: (1) Comparison of clinical outcomes of successful CTO-PCI versus CTO-MT alone (including failed CTO PCI and initial MT (CTO-PCI not attempted)) in patients with or without DM/comparison of clinical outcomes of successful CTO-PCI in patients with versus without DM. (2) They reported at least one of the following long-term (≥ 12 months) adverse outcomes: all-cause mortality, cardiac mortality, target lesion revascularization (TLR), target vessel revascularization (TVR), acute myocardial infarction (AMI), and major adverse cardiac events (MACEs). (3) Observational studies and RCTs published as full articles in the English language.

Studies were excluded according to the following criteria: (1) Only bare metal stent implantation or balloon angioplasty without drug-eluting stent (DES) implantation. (2) Other types of studies, including reviews, meta-analyses, comments, editorials, and conference abstracts. (3) Studies that were duplicates.

### Definitions, outcomes, and follow-up periods

The primary endpoint of this study was MACEs, a composite endpoint including cardiac death and/or all-cause death, MI, and/or revascularization after stent implantation. Since the data for major adverse cardiac and cerebrovascular events (MACCEs) have been reported in only 1 study from Yang et al., MACEs and MACCEs have been combined in the same category.

Secondary endpoints in this meta-analysis include all-cause death, cardiac death, MI, TLR, and TVR, which are defined according to the Academic Research Consortium [17]. If death in the eligible studies was not clearly defined whether it was cardiac or noncardiac or both, we assumed it was an all-cause death and included it in the analysis. Additionally, the CTO lesions in the present study were defined as complete blockage of a coronary artery with TIMI 0 for more than 3 months. Successful CTO-PCI was predefined as recanalization of the lesion with residual stenosis < 30% and TIMI grade ≥ 2.

### Data extraction and quality assessment

Two investigators (MLC and KSL) systematically retrieved important information and data regarding the eligible studies, baseline characteristics of the patients, and the prespecified adverse outcomes. In addition, the two investigators also assessed the quality of the observational studies and RCTs using the Newcastle–Ottawa Scale (NOS) and Revised Jadad’s Scale, respectively. Any disagreements encountered in the processes of study selection, data extraction and quality assessment were resolved by discussion with the third investigator (ZNJ).

### Statistical analysis

The heterogeneity across the studies was assessed by Cochran’s Q-test (*p* < 0.1 was regarded as statistically significant) and I^2^ statistics, which estimate heterogeneity quantitatively (I^2^ value < 25% indicates no or mild heterogeneity, 25–50% indicates moderate heterogeneity, and > 50% indicates high heterogeneity). If I^2^ > 50% or *p* < 0.1, we calculated risk ratios (RRs) with 95% confidence intervals (CIs) using the random-effects model. If I^2^ ≤ 50% or *p* ≥ 0.1, the fixed-effects model was used. Additionally, the publication bias in the included studies was assessed visually by funnel plots. All statistical analyses in the present study were performed using RevMan 5.3 software (Cochrane Collaboration; Copenhagen, Denmark), and *p* < 0.05 was considered statistically significant.

## Results

### Study selection and general characteristics of the eligible studies

**Fig. 1:**
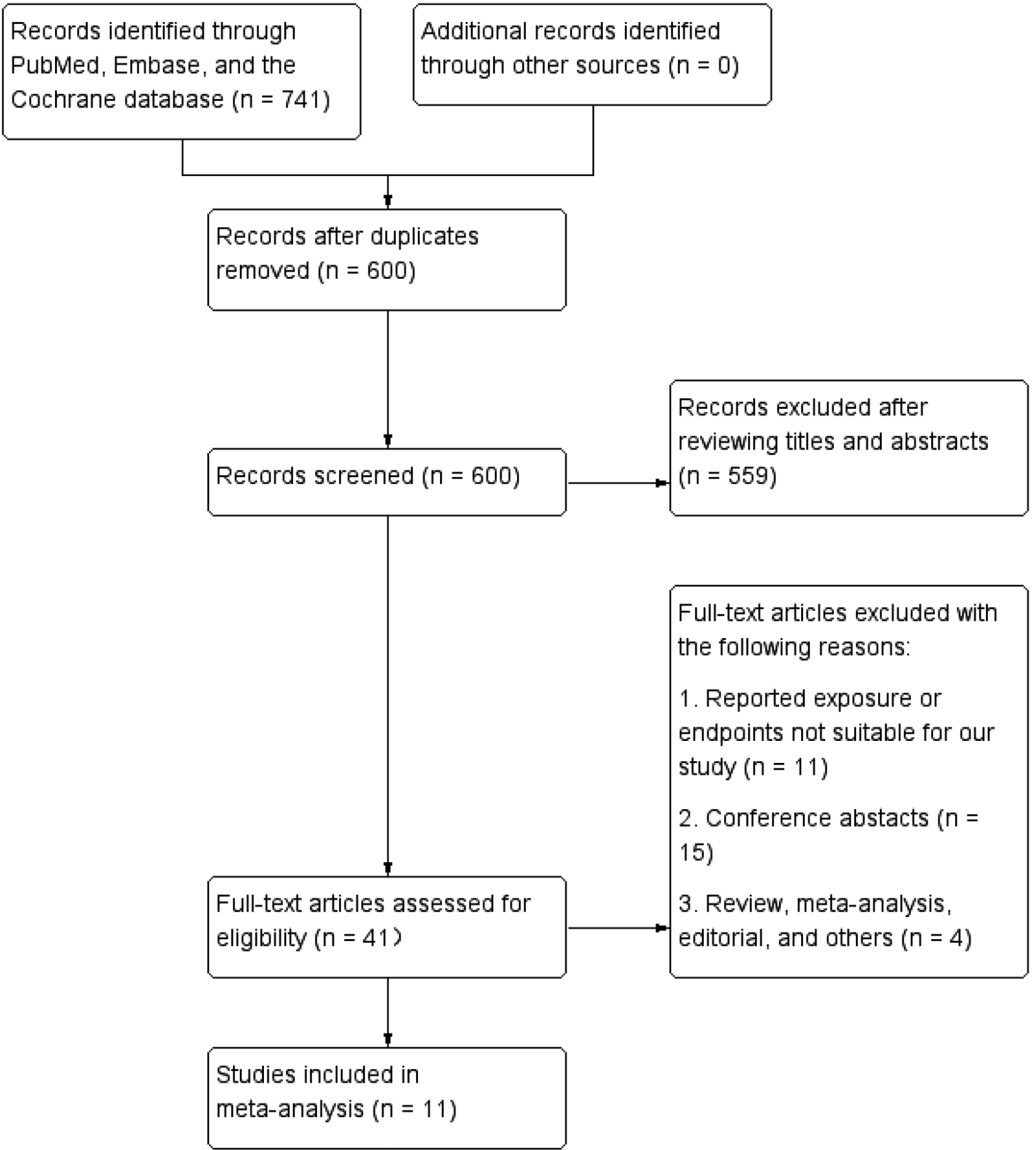
The flow diagram of study selection

A total of 741 potentially relevant studies were initially identified in the present study, of which 600 records were further screened after removing duplicates. After title and abstract screening, a total of 41 full-text articles were assessed for eligibility. Of these 41 studies, 30 records were subsequently excluded for various reasons. Finally, 11 articles were selected and included in this meta-analysis [18–28]. The flowchart of study selection is presented in Fig. 1.

The general characteristics of the 11 eligible studies are summarized in Table 1. A total of 9847 patients consisting of 4238 DM patients and 5609 non-DM patients, which were recruited from 1998 to 2018 at different medical centres, were included in this meta-analysis. The 11 studies included were all published from 2011 to 2020, and most of them were nonrandomized, observational, and comparative studies. Importantly, the 11 studies included in this meta-analysis all had good quality according to the NOS and the Revised Jadad’s Scale. The reported endpoints and follow-up periods of the eligible studies are presented in Table 2.

**Table 1:** General features of the eligible studies

Studies	Country/Region	Year	Study type/Patients enrollment period	Multicenter	Study patients	Stent	Study quality
Liu et al	Japan	2013	Respective cohort study/ 2005–2009	NO	Successful CTO PCI: DM/ Non-DM:51/102	DES:62.9%, BMS:31.4%	8
Claessen	United states, South Korea, and Italy	2011	Prospective cohort study/ 1998–2007	YES	1.Successful CTO PCI: DM/ Non-DM: 202/528 2. DM (395) Failed/successful PCI: 120/275 Non-DM (1347) Failed/successful PCI: 432/915	1.DES:100% 2.DES: more than 60%	8
Sohrabi et al	Iran	2011	Prospective cohort study/ 2009–2011	NO	Successful CTO PCI: DM/ Non-DM:34/129	DES:NA, BMS: NA	8
Ruiz-Garcia	Spain and Portugal	2015	RCT/NA	YES	Successful CTO PCI: DM/Non-DM:75/132	DES: 100%	4
Rha et al	Korea	2015	Respective cohort study/ 2007–2009	YES	Successful CTO PCI: DM/Non-DM: 920/920	DES:100%	8
Yang et al	China	2020	Prospective cohort study/ 2016–2018	NO	Successful CTO PCI: DM/Non-DM:198/335	DES:100%	8
Sanguineti et al	France	2017	Prospective cohort study/ 2004–2012	NO	DM(362): Failed /successful PCI: 103/259 Non-DM (958): Failed /successful PCI: 227/731	DES: more than 90%	9
Tsai et al	Taiwan	2020	Respective cohort study/ 2005–2015	NO	DM (313) Failed/successful PCI: 48/265 Non-DM(426) Failed/successful PCI: 72/354	NA	8
Guo et al	China	2020	Respective cohort study/ 2007–2018	NO	DM (755) PCI / initial MT: 249/506 Non-DM (1260) PCI / initial MT: 469/791	Almost all DES	8
Yan et al	China	2019	Respective cohort study/ 2007–2017	NO	DM (733) PCI/CTO-MT: 309/424	Almost all DES	7
Flores-Umanzor et al	Spain	2020	Prospective cohort study/ 2010–2014	NO	DM (402) PCI/CTO-MT: 76/326	DES:100%	8

*CTO* chronic total occlusion, *PCI* percutaneous coronary intervention, *DM* Diabetes mellitus, *DES,*drugs-eluting stent, *BMS* bare metal stent, *NA* not available, *RCT* randomized controlled trials, *MT* medical treatment. CTO-MT consists of initial MT and failed CTO-PCI

**Table 2:** Reported outcomes and follow up periods

Studies	Follow-up periods	Clinical outcomes
Liu et al	36 ± 12 months	Cardiac death, AMI, TLR, MACE
Claessen et al	5 years	All-cause mortality, MI, TVR, CABG, MACE
Sohrabi et al	12 months	Cardiac death, Non-cardiac death, MI, TVR, MACE
Ruiz-Garcia	12 months	Death, TVR, AMI, stent thrombosis, stroke, MACE
Rha et al	12 months	Total death, cardiac death, MI, TLR, TVR, TLR-MACE, TVR-MACEs, Total MACE
Yang et al	13.5 ± 4.1 months	All-cause death, cardiac death, MI, stroke, repeat revascularization, MACCE
Sanguineti et al	4.2 (2.5–6.6) years	All-cause mortality, cardiac death, TLR, Total TVR, CABG, MI, MACE
Tsai et al	5 (1–10) years	All-cause mortality, cardiovascular mortality, MI, MACE
Guo et al	2.6 (1.2–4.7) years	Cardiac mortality, MI, TVR, and MACE
Yan et al	42(24–78.25) months	All-cause death, cardiac death, MI, repeat revascularization, TVR
Flores-Umanzor et al	4.03 (2.6–4.8) years	All-cause death, cardiac death, AMI, PCI-of non-CTO vessel, heart failure hospitalization

*AMI* acute myocardial infarction, *TLR* target lesion revascularization, *MACE* major adverse cardiac events, *TVR* target vessel revascularization, *CABG* coronary artery bypass grafting, *MACCE* major adverse cardiac and cerebrovascular events, *PCI* percutaneous coronary intervention, *CTO* chronic total occlusion

### Baseline characteristics of patients enrolled in the eligible studies

As shown in Table 3, the patients in the DM group were also more likely to have a lower left ventricular ejection fraction (LVEF) and to have a history of hypertension (HTN) than patients in the non-DM group. However, compared with the DM group, men and current smokers may be more common in the non-DM group. Regarding angiographic characteristics, we discovered that the percentage of cases with multivessel disease was higher in the DM group than in the non-DM group.

**Table 3:** Baseline characteristics of DM and non-DM patients treated by successful CTO PCI

Study	Age (yrs) DM/Non-DM	Male (%) DM/Non-DM	Smoker(%) DM/Non-DM	HTN (%) DM/Non-DM	LVEF(%) DM/Non-DM	CTO location (%) LAD/LCX/RCA	MVD (%) DM/Non-DM	J-CTO DM/Non-DM	SYNTAX DM/Non-DM
Liu et al	76.58 ± 8.95/ 74.48 ± 8.1	66.7/84.5	49/67.6	78.4/67	55.31 ± 16.23/ 57.35 ± 14.5	NA	45.1/36.1	NA	NA
Sohrabi et al	58.11 ± 10.94/ 58.23 ± 11.13	64.7/80.6	20.6/36.4	58.8/38	NA	DM:55.9/17.6/23.1 Non-DM: 61.2/15.5/23.3	44.1/34.7	NA	NA
Ruiz-Garcia et al	64.9 ± 9.2/ 63.8 ± 11.0	77.3/86.4	57.3/54.5	70.7/66.7	48.4 ± 13.2/ 55.5 ± 12.8	DM:34.7/0/45.3 Non-DM:44.7/0/36.4	65.3/50.8	NA	NA
Rha et al	63.77 ± 9.29/ 64.41 ± 9.88	71.6/72.5	27.8/27.8	76.1/76.0	NA	DM:40.8/19.9/38.9 Non-DM: 36.7/19.7/43.4	NA	NA	NA
Yang et al	63.02 ± 10.36/ 62.37 ± 11.35	74/84	38/36	77/66	51.36 ± 7.46/ 53.00 ± 7.27	DM:32/15/53 Non-DM: 35/14/51	85/76	NA	NA
Claessen et al	62.0 ± 9.8/ 60.5 ± 10.9	80.2/85	42.8/36.1	70.3/62.8	52.2 ± 10.2/ 54.5 ± 9.4	DM:34.6/26.0/39.4 Non-DM: 39.2/18.5/41.8	64.5/58.0	NA	NA
Tasi et al	70 ± 12/ 66 ± 14	86/96	35/46	85/68	47 ± 12/ 50 ± 12	DM:40/26/52 Non-DM: 46/26/44	85/78	2.54 ± 1.00/ 2.31 ± 0.99	18 ± 6/ 19 ± 6
Guo et al	64.1 ± 8.9/ 62.9 ± 10.1	68.3/79.1	34.9/46.7	73.5/62.9	53.3 ± 9.1/ 54.8 ± 8.1	DM:40.2/24.5/47.4 Non-DM: 40.5/22.4/48.0	77.7/63.5	1.41 ± 0.98/ 1.47 ± 1.03	21.5 ± 7.5/ 18.9 ± 6.9
Sanguineti et al	65.5 ± 10.4/ 62.2 ± 11.6	82.3/86.7	21.8/28.6	72.8/54.0	55.68 ± 9.01/ 57.01 ± 9.57	DM:31.2/26.4/42.8 Non-DM: 31.1/21.6/47.2	64.9/59.4	1.45 ± 0.84/ 1.34 ± 0.9	NA

*DM* diabetes mellitus, *CTO* chronic total occlusion, *PCI* percutaneous coronary intervention, *HTN *hypertension, *LVEF* left ventricular ejection fraction, *LAD* left anterior descending artery, *LCX* left circumflex artery, *RCA* right coronary artery, *MVD* multivessel disease, *J-CTO* Japanese-chronic total occlusion, *NA* not available

The baseline characteristics of the DM and non-DM patients treated by successful CTO-PCI versus CTO-MT are summarized in Tables 4 and 5. Notably, patients treated by successful CTO-PCI, regardless of diabetic status, more often had CTO in the left anterior descending artery (LAD) and were less likely to develop multivessel disease than patients in the CTO-MT group. In addition, the J-CTO and SYNTAX scores seem to be lower in the successful CTO-PCI group than in the CTO-MT group based on the limited data.

**Table 4:** Baseline characteristics of DM patients treated by successful CTO-PCI versus CTO-MT

Study	Age (yrs) PCI/MT	Male (%) PCI/MT	Smoker(%) PCI/MT	HTN (%) PCI/MT	LVEF(%) PCI/MT	CTO location (%) LAD/LCX/RCA	MVD (%) PCI/MT	J-CTO PCI/MT	SYNTAX PCI/MT
Claessen et al	61.9 ± 9.5/ 62.1 ± 9.4	81.1/85.8	35.4/27.5	69.1/74.5	51.8 ± 10.0/ 50.9 ± 11.5	PCI:32.4/27.4/40.2 MT:33.1/23.3/42.9	67.4/81.7	NA/NA	NA/NA
Guo et al	64.1 ± 8.9/ 65.6 ± 10.2	68.3/70.6	34.9/37.2	73.5/76.9	53.3 ± 9.1/ 51.4 ± 11.1	PCI:40.2/24.5/47.4 MT:27.2/36.0/51.2	77.7/86.4	1.41 ± 0.98/ 1.78 ± 1.23	21.5 ± 7.5/ 24.6 ± 9.1
Tsai et al	70 ± 12/ 70 ± 13	86/88	35/35	85/88	47 ± 12/ 47 ± 12	PCI:40/26/52 MT:33/33/63	85/96	2.54 ± 1.00/ 2.66 ± 1.00	18 ± 6/ 20 ± 7
Yan et al	59.06 ± 9.03/ 60.65 ± 10.58	79.3/72.7	55.3/48.8	66.7/69.5	62.0(57.0–66.0)/ 61.0(55.0–66.0)	PCI:0/0/100/0 MT:0/0/100/0	71.2/74.1	NA/NA	19(12–21)/ 20(13–27)
Flores-Umanzor et al	66.8 ± 10.2/ 70.2 ± 10.4	83/79	55/52	82/85	46.9 ± 13.1/ 43.0 ± 14.1	PCI:35/16/38 MT:18/19/52	82/87	NA/NA	22.7 ± 10.5/ 24.3 ± 12.3
Sanguineti et al	65.5 ± 10.4	82.3	21.8	72.8	55.68 ± 9.01	31.2/25.4/42.8/0.6	64.4	1.45 ± 0.84	NA

*DM* diabetes mellitus, *CTO* chronic total occlusion, *PCI* percutaneous coronary intervention, *MT* medical treatment, *HTN* hypertension, *LVEF* left ventricular ejection fraction, *LAD* left anterior descending artery, *LCX* left circumflex artery, *RCA* right coronary artery, *MVD* multivessel disease, *J-CTO* Japanese-chronic total occlusion, *NA* not available

**Table 5:** Baseline characteristics of non-DM patients treated by successful-CTO PCI versus CTO-MT

Study	Age (yrs) PCI/MT	Male (%) PCI/MT	Smoker(%) PCI/MT	HTN (%) PCI/MT	LVEF(%) PCI/MT	CTO location (%) LAD/LCX/RCA	MVD (%) PCI/MT	J-CTO PCI/MT	SYNTAX PCI/MT
Claessen et al	60.9 ± 11.0/ 62.1 ± 10.7	85.9/89.8	25.8/21.8	57.2/55.1	54.3 ± 10.1/ 53.0 ± 10.5	PCI:37.7/21.9/39.9 MT:30.4/22.2/46.9	64.6/74.0	NA/NA	NA/NA
Guo et al	62.9 ± 10.1/ 64.3 ± 11.0	79.1/82.2	46.7/46.0	62.9/64.2	54.8 ± 8.1 52.3 ± 9.6	PCI:40.5/22.4/48.0 MT:33.1/30.7/47.9	63.5/86.3	1.47 ± 1.03/ 1.80 ± 1.24	18.9 ± 6.9/ 22.6 ± 8.8
Tsai et al	66 ± 14/ 68 ± 13	96/90	46/42	68/75	50 ± 12/ 48 ± 14	PCI:46/26/44 MT:42/29/46	78/83	2.31 ± 0.99/ 2.40 ± 0.89	19 ± 6/ 20 ± 7
Sanguineti et al	62.2 ± 11.6	86.7	28.6	54.0	57.1 ± 9.57	31.1/21.6/47.2/0.1	59.4	1.34 ± 0.9	NA

*DM* diabetes mellitus, *CTO* chronic total occlusion, *PCI* percutaneous coronary intervention, *MT* medical treatment, *HTN* hypertension, *LVEF* left ventricular ejection fraction, *LAD* left anterior descending artery, *LCX* left circumflex artery, *RCA* right coronary artery, *MVD* multivessel disease, *J-CTO* Japanese-chronic total occlusion, *NA* not available

### Successful CTO-PCI versus CTO-MT for clinical outcomes in patients with DM

**Fig. 2:**
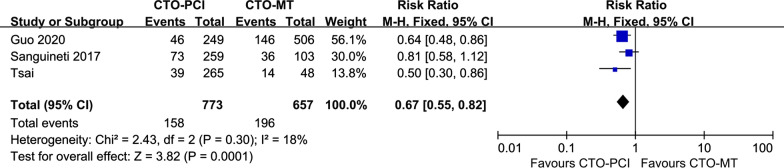
Forest plot comparing MACEs between successful CTO-PCI and CTO-MT in DM patients. *MACEs* major adverse cardiac events, *CTO* chronic total occlusions, *PCI* percutaneous coronary intervention, *MT* medical treatment, *DM* diabetes mellitus

**Fig. 3:**
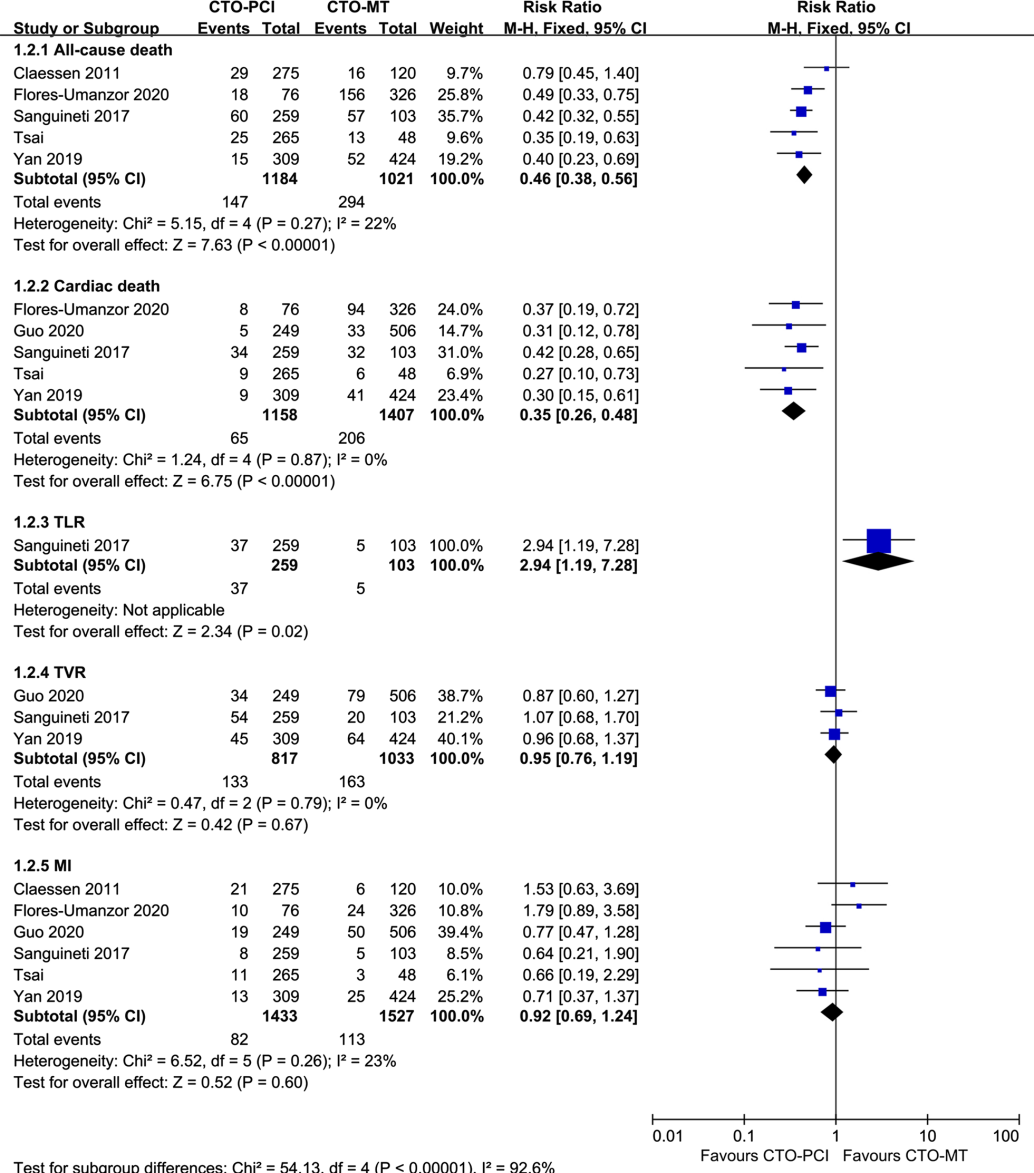
Forest plot comparing secondary endpoints between successful CTO-PCI and CTO-MT in DM patients. *CTO* chronic total occlusions, *PCI* percutaneous coronary intervention, *MT,*medical treatment, *DM* diabetes mellitus

A total of 6 studies were included to compare successful CTO-PCI versus CTO-MT for clinical outcomes in DM patients [18, 23, 24, 26–28], and 3 of 6 eligible studies reported the primary endpoint MACEs [18, 23, 27]. As revealed in Fig. 2, our pooled results demonstrated that the incidence of MACEs was significantly lower in the successful CTO-PCI group than in the CTO-MT group (RR = 0.67, 95% CI 0.55–0.82, *p* = 0.0001). When considering the secondary endpoints, as presented in Fig. 3, the successful CTO-PCI group also had a lower risk of all-cause death (RR = 0.46, 95% CI 0.38–0.56, *p* < 0.00001) and cardiac death (RR = 0.35, 95% CI 0.26–0.48, *p* < 0.00001). However, for other secondary endpoints (TVR and MI) in DM patients, no notable difference was observed between patients treated with successful CTO-PCI and CTO-MT.

Subgroup analysis comparing successful CTO-PCI with failed CTO-PCI demonstrated that CTO patients with DM could obtain more benefits from successful CTO-PCI in terms of MACEs (RR = 0.71, 95% CI 0.54–0.94, *p* = 0.02), all-cause death (RR = 0.47, 95% CI 0.37–0.58, *p* < 0.00001) and cardiac death (RR = 0.38, 95% CI 0.26–0.54, *p* < 0.00001). Additionally, as shown in Additional file 1: Table S1, successful CTO-PCI may also be better than initial MT in reducing the risk of MACEs (RR = 0.64, 95% CI 0.48–0.86, *p* = 0.003), all-cause death (RR = 0.36, 95% CI 0.20–0.66, *p* = 0.00009), and cardiac death (RR = 0.29, 95% CI 0.16–0.53, *p* < 0.0001).

### Successful CTO-PCI versus CTO-MT for clinical outcomes in patients without DM

**Fig. 4:**
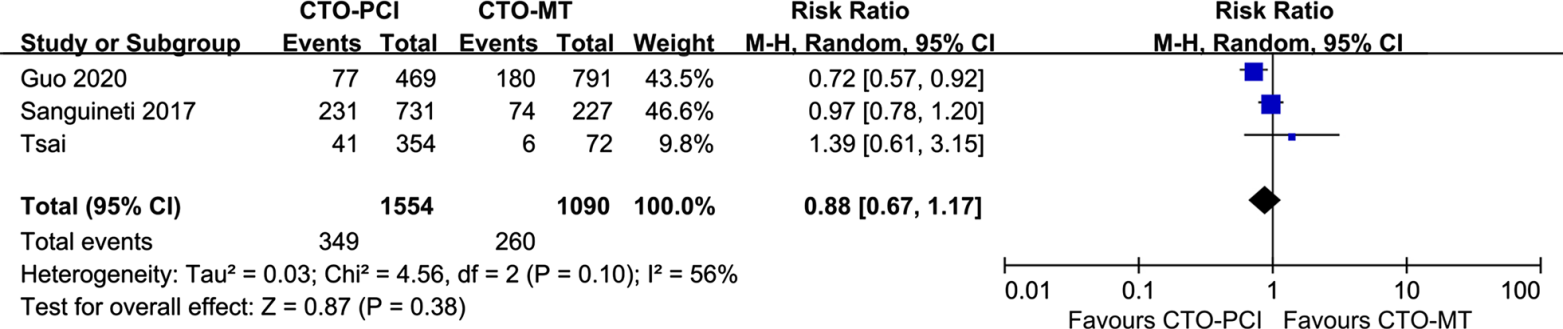
Forest plot comparing MACEs between successful CTO-PCI and CTO-MT in non-DM patients. *MACEs* major adverse cardiac events, *CTO* chronic total occlusions, *PCI* percutaneous coronary intervention, *MT* medical treatment, *DM* diabetes mellitus

**Fig. 5:**
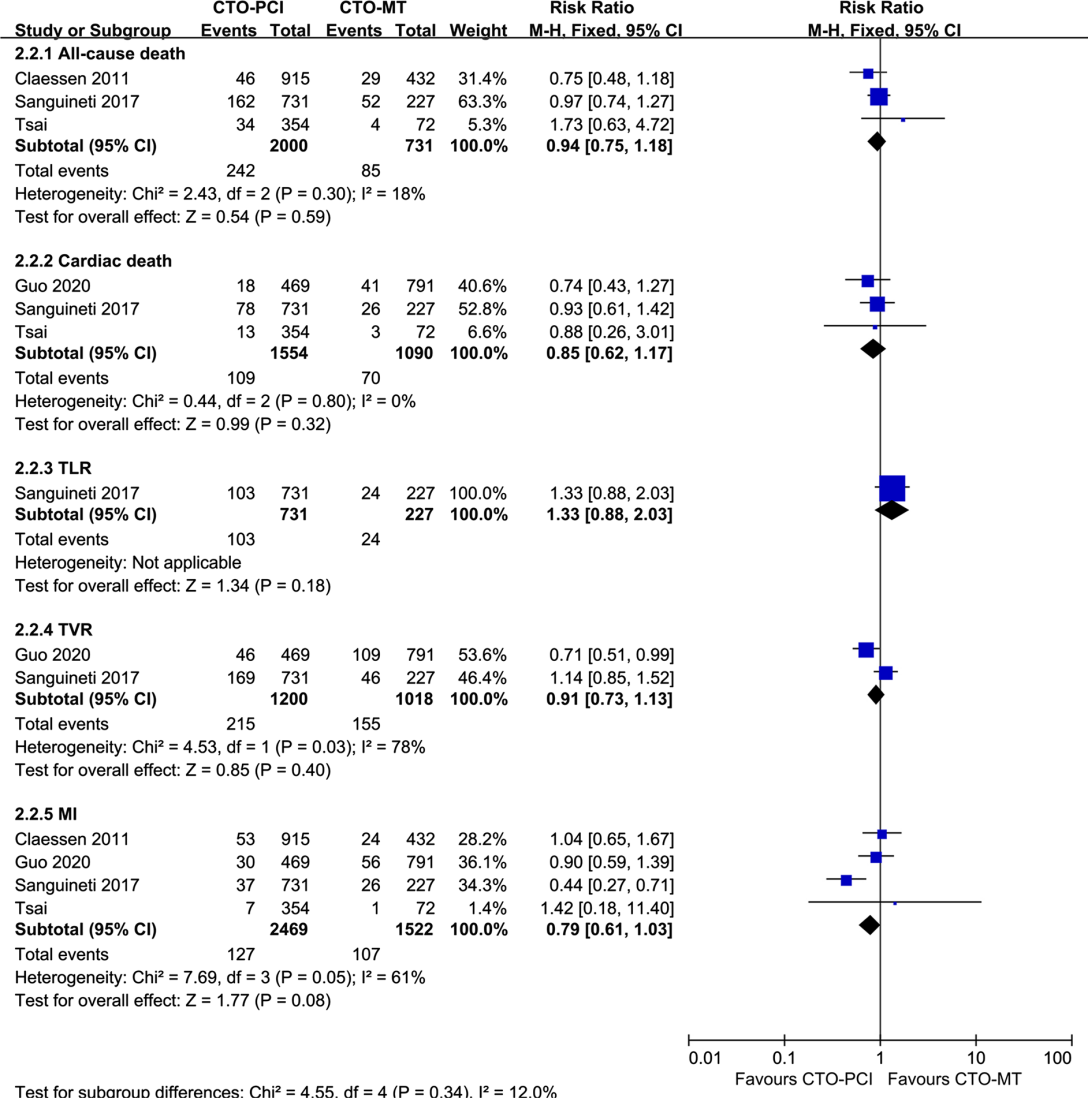
Forest plot comparing secondary endpoints between successful CTO-PCI and CTO-MT in non-DM patients. *CTO* chronic total occlusions, *PCI* percutaneous coronary intervention, *MT* medical treatment, *DM* diabetes mellitus

Four studies were included in this meta-analysis to compare successful CTO-PCI versus CTO-MT for clinical outcomes in non-DM patients [18, 23, 27, 28]. As indicated in Fig. 4, the prevalence of MACEs was comparable between the successful CTO-PCI group and the CTO-MT group in non-DM patients (RR = 0.88, 95% CI 0.67–1.17, *p* = 0.38). Additionally, as presented in Fig. 5, the secondary endpoints, including all-cause death, cardiac death, TLR, TVR, and MI, were also similar between the successful CTO-PCI group and the CTO-MT group in non-DM patients.

As indicated in Additional file 1: Table S2, another subgroup analysis demonstrated that in non-DM patients, there were no significant differences between successful CTO-PCI and failed CTO-PCI in the incidence of MACEs (RR = 1.00, 95% CI 0.81–1.24, *p* = 0.97) or other secondary endpoints. Furthermore, Guo et al. reported in their study (the only study comparing successful CTO-PCI versus initial MT in non-DM patients) that the long-term prognosis of initial MT was comparable to successful CTO-PCI in non-DM patients [23].

### Successful CTO-PCI for clinical outcomes in patients with versus without DM

Subsequently, we systematically compared the long-term prognosis of successful CTO-PCI in patients with versus without DM. A total of 9 studies were included to analyse this issue [18–23, 25, 27, 28], and 4 of them reported a more than 3-year follow-up period[18, 22, 27, 28]. As presented in the supplementary material (Additional File 1: Fig. S1), for the patients undergoing successful CTO-PCI in the DES era, the rate of MACEs was significantly higher in the DM group than in the non-DM group (RR = 1.26, 95% CI 1.02–1.56, *p* = 0.03).

**Fig. 6:**
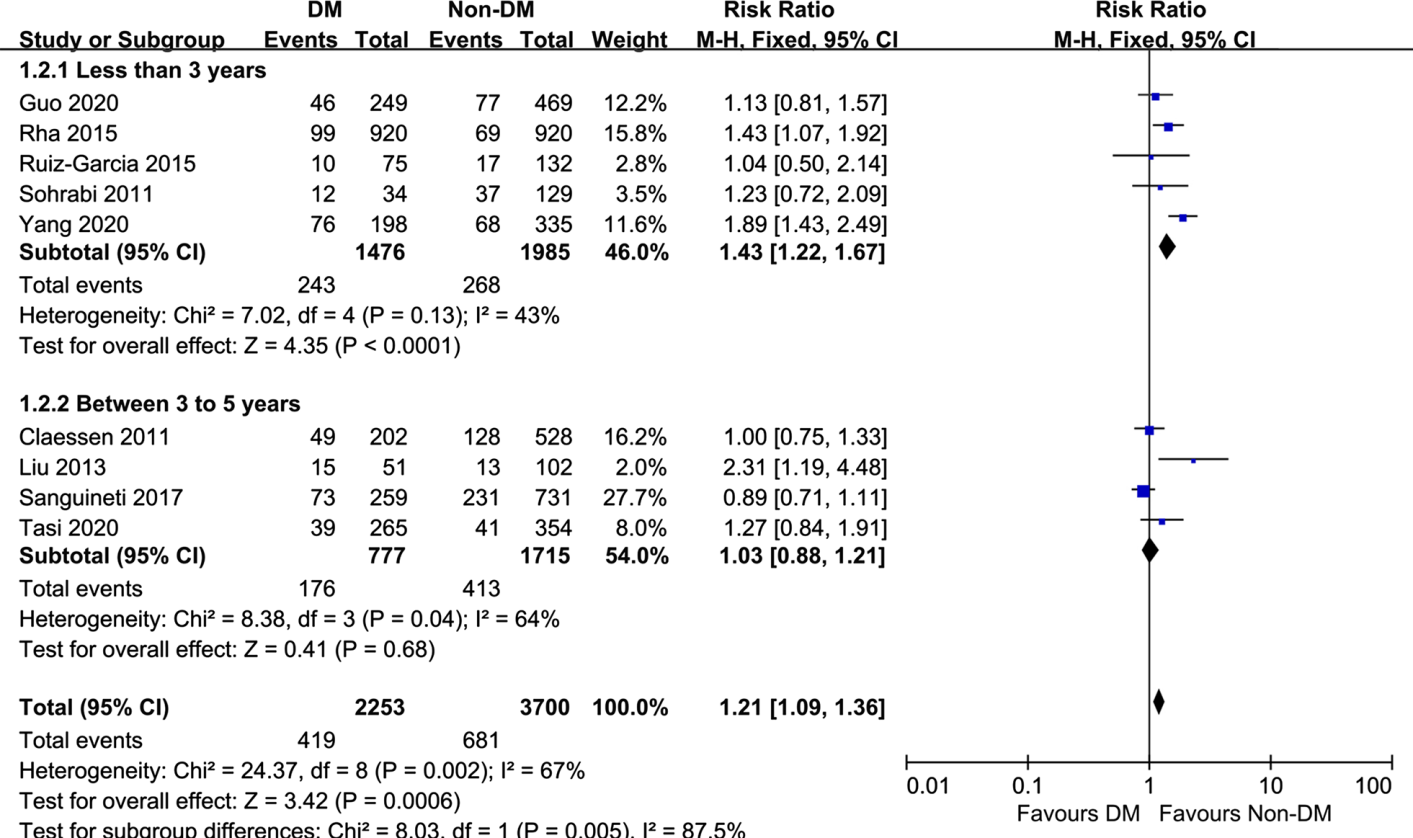
Subgroup analysis of the rate of MACEs following successful CTO-PCI in patients with versus without DM. *MACEs* major adverse cardiac events, *CTO* chronic total occlusions, *PCI* percutaneous coronary intervention, *DM* diabetes mellitus

Subgroup analysis of the MACE rate by follow-up period was performed to explore the sources of heterogeneity. The subgroup analysis demonstrated that the DM group had a higher rate of MACEs than the non-DM group (RR = 1.43, 95% CI 1.22–1.67, *p* < 0.0001, Fig. 6) within 3 years after successful CTO-PCI. However, no significant difference was observed in the subgroup with a follow-up period longer than 3 years (RR = 1.14, 95% CI 0.85–1.53, *p* = 0.39).

**Fig. 7:**
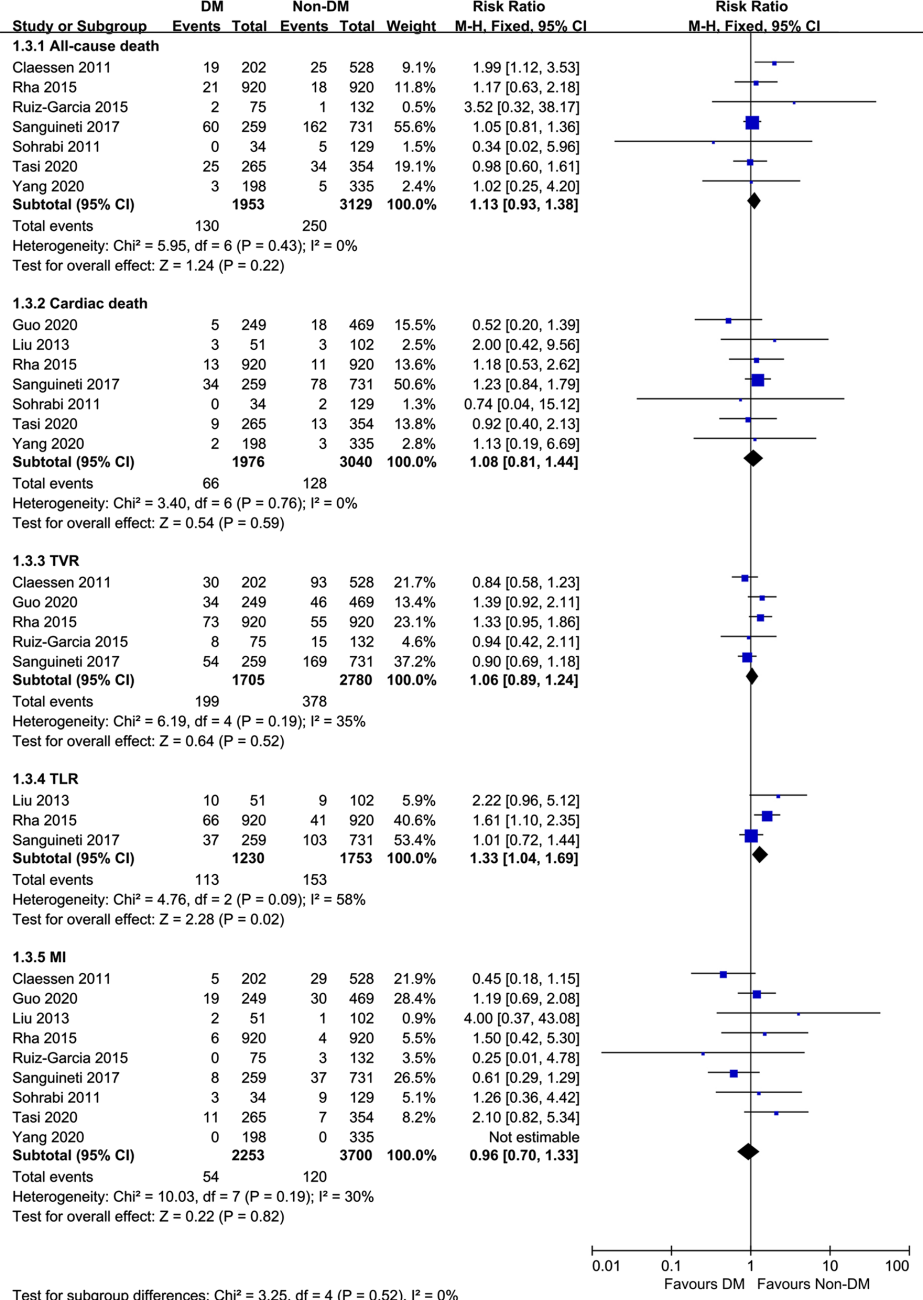
Forest plot comparing secondary endpoints following successful CTO-PCI in patients with versus without DM. *CTO* chronic total occlusions, *PCI* percutaneous coronary intervention, *DM* diabetes mellitus

For secondary endpoints, as we can see from Fig. 7, there were no significant differences between the DM and non-DM groups in secondary endpoints: all-cause death (RR = 1.13, 95% CI 0.93–1.38, *p* = 0.22), cardiac death (RR = 1.08, 95% CI 0.81–1.44, *p* = 0.59), TVR (RR = 1.06, 95% CI 0.89–1.24, *p* = 0.52), TLR (RR = 1.40, 95% CI 0.92–2.13, *p* = 0.11) and MI (RR = 0.96, 95% CI 0.70–1.33, *p* = 0.82).

### Publication bias and sensitivity analysis

Based on a visual inspection of funnel plots (Additional file 1: Figs S2, S3, S4), there was no obvious evidence of publication bias for the selected studies assessing successful CTO-PCI outcomes in patients with versus without DM. However, funnel plots for the studies investigating successful CTO-PCI versus CTO-MT for outcomes in patients with or without DM were not drawn due to the limited number of studies. Notably, the primary results of this meta-analysis remained stable after the sensitivity analysis.

## Discussion

The results from this meta-analysis of 11 studies with 9847 patients demonstrated 3 important findings. First, the successful CTO-PCI group suffered a lower risk of MACEs, all-cause death, and cardiac death than the CTO-MT group for the DM patients. Second, successful CTO-PCI was not associated with reduced MACEs or other secondary endpoints compared to CTO-MT in the non-DM group. Third, for patients undergoing CTO-PCI successfully in the DES era, the non-DM group may be associated with a lower prevalence of MACEs than the DM group, especially within the first 3 years post PCI.

### Successful CTO-PCI versus CTO-MT in patients with or without DM

Given the considerable progress made in recent years, PCI has become a safe and important treatment option for patients with CTO [1]. According to previous studies, approximately 40% of the patients undergoing CTO-PCI also had DM [10, 29]. Although CTO lesions are more complex in DM patients, 2 recent large CTO studies, in which contemporary dedicated equipment and skills, including hybrid algorithms, have been applied, reported that procedural success and the prevalence of periprocedural complications were acceptable and comparable in patients with versus without DM [12, 30]. However, the long-term prognosis of CTO revascularization with PCI in DM patients remains controversial, and few RCTs have been designed to assess it specifically.

Our present meta-analysis of 6 observational studies demonstrated that successful CTO-PCI might be associated with improved clinical outcomes compared to CTO-MT alone in DM patients. The conclusion obtained from our meta-analysis is not consistent with the findings of the RCT COURAGE, which demonstrated that there was no obvious difference in the long-term prognosis between the PCI group and the MT group in DM patients with stable coronary disease [31]. This discrepant finding may be explained by the following reasons. First, COURAGE was not performed in the CTO setting. Second, most of the patients recruited in our study received DES, while only 3% of COURAGE participants were treated with DES. Third, approximately 30% of patients in the MT group crossed over to revascularization during the follow-up period, which may underestimate the actual effect of successful PCI in patients with stable coronary disease.

For non-DM patients, our present study reveals that successful CTO-PCI may not be associated with a better prognosis in the long term compared to CTO-MT alone, which is consistent with previous studies [23, 27]. However, the reasons behind the phenomenon that only the DM group, instead of the non-DM group, could benefit from CTO revascularization remain unclear. We speculate that it may be associated with coronary collateral circulation, which mainly supplies blood perfusion downstream of the CTO. DM is strongly associated with poor collateral circulation [13, 14, 21], and therefore, successful CTO-PCI may significantly improve blood perfusion downstream of the CTO in DM patients.

### Outcomes of successful CTO-PCI in patients with versus without DM

Regarding the long-term prognosis of successful CTO-PCI in patients with versus without DM, a recent meta-analysis by Wang et al. revealed that compared with CTO patients without DM, the prevalence of MACEs, mortality, and repeat revascularization were all higher in patients with DM [32]. Consistent with the findings of Wang and co-workers, our study also demonstrated that the rate of MACEs after successful CTO-PCI was higher in DM patients than in non-DM patients, especially in the subgroup with a follow-up period of less than 3 years. In contrast, through subanalysis of the data from the RCT CLBELES, Ruiz Garcia et al. reported that the rate of MACEs following successful CTO-PCI was comparable between DM and non-DM patients in the DES era [19]. However, the study of Ruiz Garcia did not accurately reflect the long-term prognosis following successful CTO-PCI in DM versus non-DM patients because of the limited sample size (207 patients consisting of 75 DM patients and 132 non-DM patients) and the relatively short follow-up period (12 months).

According to previous studies, our findings that CTO patients with DM have a worse long-term prognosis than non-DM patients after successful CTO-PCI may be explained by the following reasons. First, DM, as a well-established risk factor for CAD, is associated with more complex angiographic and clinical characteristics [8, 12]. Second, DM could confer a greater risk for adverse outcomes by exerting a detrimental effect on glucose and lipid metabolism and vascular endothelial function [33–36]. Third, poor coronary collateralization, which is frequently associated with DM, may also partially contribute to an unfavourable prognosis [13, 37].

## Limitations

Some limitations should be acknowledged in this meta-analysis. First, most of the studies included in our meta-analysis were observational studies, and some of the studies cited included only subgroups of relevant patients; therefore, we should be cautious when interpreting the results. Second, our study combined failed CTO-PCI and initial CTO-MT as the CTO-MT group, which may limit the validity of the conclusions. Additionally, our present study failed to explore the long-term prognosis of the initial PCI strategy versus the initial optimal MT in CTO patients with or without DM. Finally, the data in our study are mostly from high-volume CTO-PCI centres, so our conclusion may not be generalizable to other cardiac centres with less experience.

## Conclusions

Based on the limited data, our meta-analysis concluded that successful CTO-PCI may be superior to CTO-MT alone for the treatment of CTO in DM patients but not in non-DM patients. Compared to CTO patients with DM, non-DM patients may have a reduced risk of MACEs, especially within 3 years after successful CTO-PCI. To verify the findings obtained from our study, multicentre RCTs with large sample sizes are warranted.

## Supplementary Information

Additional file1: Table S1: Subgroup analysis comparing successful CTO-PCI versus failed CTO-PCI, initial MT in DM patients. Table S2: Subgroup analysis comparing successful CTO-PCI versus failed CTO-PCI, initial MT in non-DM patients. Figure S1: Forest plot comparing MACEs following successful CTO-PCI in patients with versus without DM. MACEs, major adverse cardiac events; CTO, chronic total occlusions; PCI, percutaneous coronary intervention; DM, diabetes mellitus. Figure S2: Funnel plot assessing the publica bias of the studies reporting risk of MACEs following successful CTO-PCI in patients with or without DM. MACEs, major adverse cardiac events; CTO, chronic total occlusions; PCI, percutaneous coronary intervention; DM, diabetes mellitus. Figure S3 Funnel plot assessing the publica bias of the studies reporting risk of MACEs following successful CTO-PCI in patients with or without DM in the subgroups. MACEs, major adverse cardiac events; CTO, chronic total occlusions; PCI, percutaneous coronary intervention; DM, diabetes mellitus. Figure S4 Funnel plot assessing the publica bias of studies reporting the risk of secondary endpoints following successful CTO-PCI in patients with versus without DM. MACEs, major adverse cardiac events; CTO, chronic total occlusions; PCI, percutaneous coronary intervention; DM, diabetes mellitus.

## Data Availability

All data generated or analyzed during this study are included in this published article.

## Abbreviations

CTO: Coronary chronic total occlusion
TIMI: Thrombolysis in myocardial infarction
PCI: Percutaneous coronary intervention
MT: Medical treatment
DM: Diabetes mellitus
CAD: Coronary artery disease
J-CTO: Japanese-chronic total occlusion
RCTs: Randomized controlled trials
TLR: Target lesion revascularization
TVR: Target vessel revascularization
AMI: Acute myocardial infarction
MACE: Major adverse cardiac events
DES: Drug-eluting stents
MACCE: Major adverse cardiac and cerebrovascular events
NOS: Newcastle–Ottawa Scale
LVEF: Left ventricular ejection fraction
HTN: Hypertension
LAD: Left anterior descending artery
