# Supplementary material for: Long-term prognosis of chronic total occlusion treated by successful percutaneous coronary intervention in patients with or without diabetes mellitus: a systematic review and meta-analysis
Source: Cardiovasc Diabetol. 2021 Jan 30;20:29. doi: 10.1186/s12933-021-01223-8 (PMC7847176; doi:10.1186/s12933-021-01223-8)

1. The details of search strategy

**PubMed** (120)

((((((((((diabetes mellitus[Title/Abstract]) OR (diabetes[Title/Abstract])) OR (type 2 diabetes mellitus[Title/Abstract])) OR (diabetes mellitus, type 2[Title/Abstract])) OR (T2DM[Title/Abstract])) OR (type 1 diabetes mellitus[Title/Abstract])) OR (diabetes mellitus, type 1[Title/Abstract])) OR (T1DM[Title/Abstract])) OR (DM[Title/Abstract])) AND (((((chronic total occlusion[Title/Abstract]) OR (chronic total coronary occlusion[Title/Abstract])) OR (chronic total occlusions[Title/Abstract])) OR (chronic total coronary occlusions[Title/Abstract])) OR (CTO[Title/Abstract]))) AND ((((((((((((((Percutaneous Coronary Intervention[Title/Abstract]) OR (Coronary Intervention, Percutaneous[Title/Abstract])) OR (Coronary Interventions, Percutaneous[Title/Abstract])) OR (Intervention, Percutaneous Coronary[Title/Abstract])) OR (Interventions, Percutaneous Coronary[Title/Abstract])) OR (Percutaneous Coronary Interventions[Title/Abstract])) OR (Percutaneous Coronary Revascularization[Title/Abstract])) OR (Coronary Revascularization, Percutaneous[Title/Abstract])) OR (Coronary Revascularizations, Percutaneous[Title/Abstract])) OR (Percutaneous Coronary Revascularizations[Title/Abstract])) OR (Revascularization, Percutaneous Coronary[Title/Abstract])) OR (Revascularizations, Percutaneous Coronary[Title/Abstract])) OR (Percutaneous coronary angioplasty[Title/Abstract])) OR (PCI[Title/Abstract]))

**Cochrane Library (41)**

#1 (diabetes mellitus):ti,ab,kw OR (diabetes):ti,ab,kw OR (type 2 diabetes mellitus):ti,ab,kw OR (diabetes mellitus, type 2):ti,ab,kw OR (T2DM):ti,ab,kw 79289

#2 (type 1 diabetes mellitus):ti,ab,kw OR (diabetes mellitus, type 1):ti,ab,kw OR (T1DM):ti,ab,kw OR (DM):ti,ab,kw 40448

#3 #1 OR #2 87360

#4 (chronic total occlusion):ti,ab,kw OR (chronic total coronary occlusion):ti,ab,kw OR (chronic total occlusions):ti,ab,kw OR (chronic total coronary occlusions):ti,ab,kw OR (CTO):ti,ab,kw 625

#5 (percutaneous coronary intervention):ti,ab,kw OR (coronary intervention, percutaneous):ti,ab,kw OR (coronary interventions, percutaneous):ti,ab,kw OR (intervention, percutaneous coronary):ti,ab,kw OR (interventions, percutaneous coronary):ti,ab,kw 10483

#6 (percutaneous coronary interventions):ti,ab,kw OR (percutaneous coronary revascularization):ti,ab,kw OR (coronary revascularization, percutaneous):ti,ab,kw OR (coronary revascularizations, percutaneous):ti,ab,kw OR (percutaneous coronary revascularizations):ti,ab,kw 4634

#7 (revascularization, percutaneous coronary):ti,ab,kw OR (revascularization, percutaneous coronary):ti,ab,kw OR (percutaneous coronary angioplasty):ti,ab,kw OR (PCI):ti,ab,kw 11569

#8 #5 OR #6 OR #7 13860

#9 #3 AND #4 AND #8 41

**Embase** (580)

#4. #1 AND #2 AND #3 580

#3. 'percutaneous coronary intervention':ab,ti OR 80,722

'coronary intervention, percutaneous':ab,ti OR

'coronary interventions, percutaneous':ab,ti OR

'intervention, percutaneous coronary':ab,ti OR

'interventions, percutaneous coronary':ab,ti OR

'percutaneous coronary interventions':ab,ti OR

'percutaneous coronary revascularization':ab,ti

OR 'coronary revascularization,

percutaneous':ab,ti OR 'coronary

revascularizations, percutaneous':ab,ti OR

'percutaneous coronary revascularizations':ab,ti

OR 'revascularization, percutaneous

coronary':ab,ti OR 'revascularizations,

percutaneous coronary':ab,ti OR 'percutaneous

coronary angioplasty':ab,ti OR 'pci':ab,ti

#2. 'chronic total occlusion':ab,ti OR 'chronic total 7,546

coronary occlusion':ab,ti OR 'chronic total

occlusions':ab,ti OR 'chronic total coronary

occlusions':ab,ti OR 'cto':ab,ti

#1. 'diabetes mellitus':ab,ti OR 'diabetes':ab,ti OR 838,544

'type 2 diabetes mellitus':ab,ti OR 'diabetes

mellitus, type 2':ab,ti OR 't2dm':ab,ti OR 'type

1 diabetes mellitus':ab,ti OR 'diabetes

mellitus, type 1':ab,ti OR 't1dm':ab,ti OR

'dm':ab,ti

1. **Supplementary table**

Supplementary table 1. Subgroup analysis comparing successful CTO-PCI versus failed CTO-PCI, initial MT in DM patients

| **Successful CTO-PCI vs failed CTO-PCI** | No. of studies | No. of participants | RR (95% CI) | P for heterogeneity | I^2^(%) | P for test |
| --- | --- | --- | --- | --- | --- | --- |
| MACEs | 2 | 675 | 0.71 (0.54, 0.94) | 0.14 | 54 % | 0.02 |
| All-cause death | 4 | 1570 | 0.47 (0.37,0.58) | 0.19 | 38 % | <0.00001 |
| Cardiac death | 3 | 1175 | 0.38 (0.26,0.54) | 0.66 | 0 % | <0.00001 |
| TLR | 1 | 362 | 2.94 (1.19, 7.28) | - | - | 0.02 |
| TVR | 2 | 862 | 0.91 (0.67,1.23) | 0.33 | 0 % | 0.52 |
| MI | 4 | 1570 | 0.97 (0.60, 1.56) | 0.58 | 0 % | 0.89 |
| **Successful CTO-PCI vs initial MT** | No. of studies | No. of participants | RR (95% CI) | P for heterogeneity | I^2^(%) | P for test |
| MACEs | 1 | 755 | 0.64 (0.48, 0.86) | - | - | 0.003 |
| All-cause death | 1 | 542 | 0.36 (0.20,0.66) | - | - | 0.0009 |
| Cardiac death | 2 | 1297 | 0.29 (0.16, 0.53) | 0.89 | 0 % | <0.0001 |
| TLR | 0 |  |  |  |  |  |
| TVR | 2 | 1297 | 0.99 (0.75, 1.31) | 0.32 | 0 % | 0.94 |
| MI | 2 | 1297 | 0.72 (0.47, 1.08) | 0.60 | 0 % | 0.11 |

CTO, chronic total occlusion; PCI, percutaneous coronary intervention; MT, medical treatment; DM, diabetes mellitus; MACEs, major adverse cardiac events; TLR, target lesion revascularization; TVR, target vessel revascularization; MI, myocardial infarction.

Supplementary table 2 . Subgroup analysis comparing successful CTO-PCI versus failed CTO-PCI, initial MT in non-DM patients

| **Successful CTO-PCI vs failed CTO-PCI** | No. of studies | No. of participants | RR (95% CI) | P for heterogeneity | I^2^(%) | P for test |
| --- | --- | --- | --- | --- | --- | --- |
| MACEs | 2 | 1384 | 1.00 (0.81, 1.24) | 0.40 | 0 % | 0.97 |
| All-cause death | 3 | 2731 | 0.94 (0.75, 1.18) | 0.30 | 18 % | 0.59 |
| Cardiac death | 2 | 1384 | 0.93 (0.62, 1.38) | 0.93 | 0 % | 0.70 |
| TLR | 1 | 958 | 1.33 (0.88, 2.03) | - | - | 0.18 |
| TVR | 1 | 958 | 1.14 (0.85, 1.52) | - | - | 0.37 |
| MI | 3 | 2731 | 0.73 (0.35, 1.54) | 0.03 | 71% | 0.41 |
| **Successful CTO-PCI vs initial MT** | No. of studies | No. of participants | Adjust HR (95% CI) from the original study of Guo et al. | | | |
| MACEs | 1 | 1260 | [77/469 vs 180/791, 0.85 (0.64, 1.15), p = 0.294] | | | |
| All-cause death | 0 | - | - | | | |
| Cardiac death | 1 | 1260 | [18/469 vs 41/791, 0.94 (0.51, 1.70), p = 0.825] | | | |
| TLR | 0 | - | - | | | |
| TVR | 1 | 1260 | [46/469 vs 109/791, 0.85(0.59, 1.22), p = 0.389] | | | |
| MI | 1 | 1260 | [30/469 vs 56/791, 1.01 (0.62, 1.65), p = 0.959] | | | |

CTO, chronic total occlusion; PCI, percutaneous coronary intervention; MT, medical treatment; DM, diabetes mellitus; MACEs, major adverse cardiac events; TLR, target lesion revascularization; TVR, target vessel revascularization; MI, myocardial infarction.

1. **Supplementary Figure**

Supplementary Figure 1

Forest plot comparing MACEs following successful CTO-PCI in patients with versus without DM. MACEs, major adverse cardiac events; CTO, chronic total occlusions; PCI, percutaneous coronary intervention; DM, diabetes mellitus.


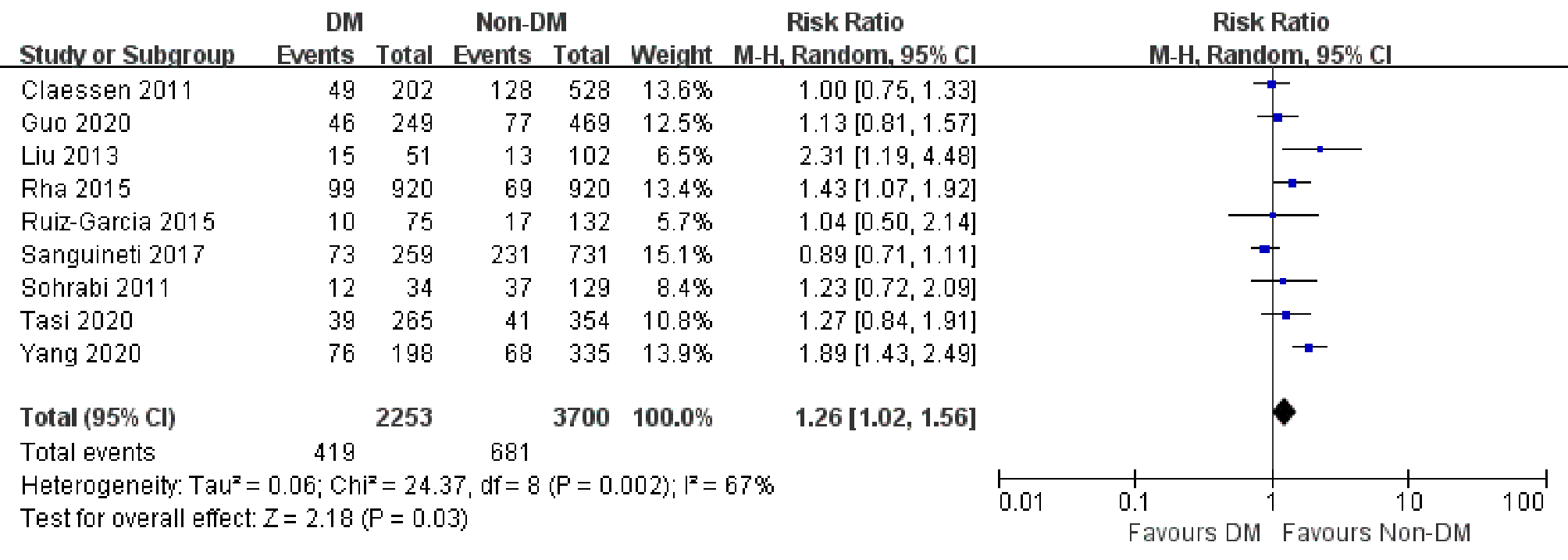


Supplementary Figure 2

Funnel plot assessing the publica bias of the studies reporting risk of MACEs following successful CTO-PCI in patients with or without DM. MACEs, major adverse cardiac events; CTO, chronic total occlusions; PCI, percutaneous coronary intervention; DM, diabetes mellitus.


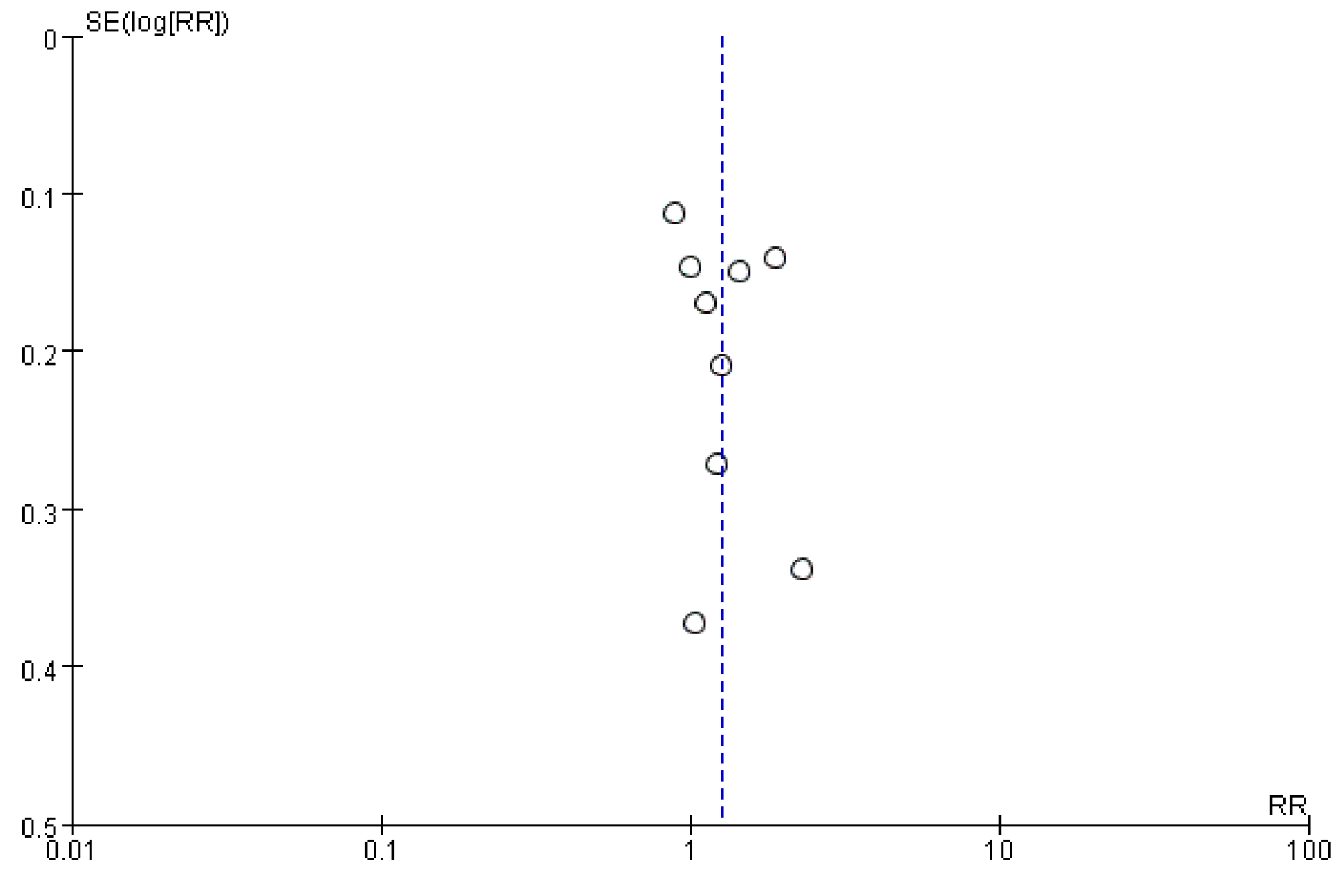


Supplementary Figure 3

Funnel plot assessing the publica bias of the studies reporting risk of MACEs following successful CTO-PCI in patients with or without DM in the subgroups. MACEs, major adverse cardiac events; CTO, chronic total occlusions; PCI, percutaneous coronary intervention; DM, diabetes mellitus.


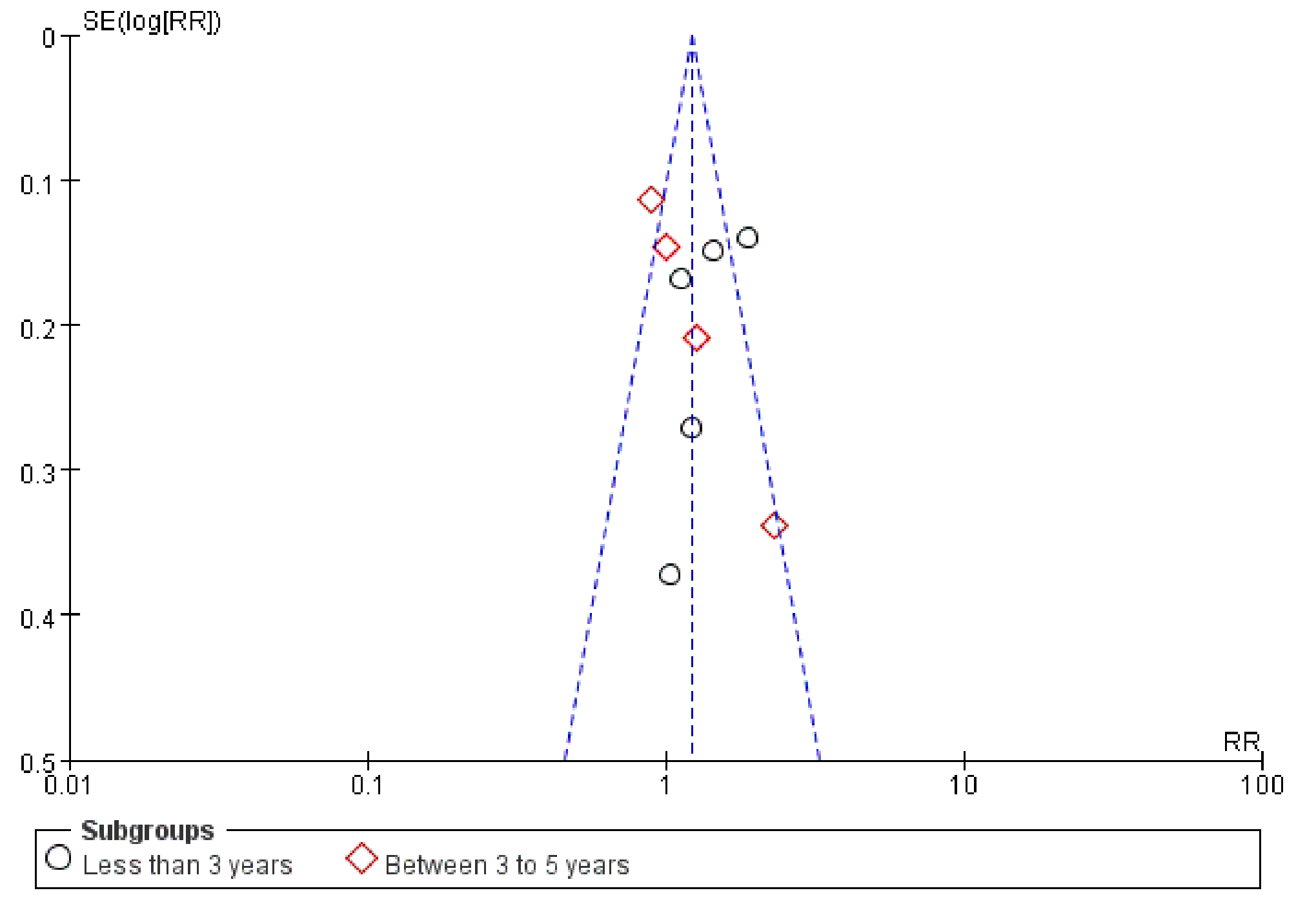


Supplementary Figure 4

Funnel plot assessing the publica bias of studies reporting the risk of secondary endpoints following successful CTO-PCI in patients with versus without DM. MACEs, major adverse cardiac events; CTO, chronic total occlusions; PCI, percutaneous coronary intervention; DM, diabetes mellitus.


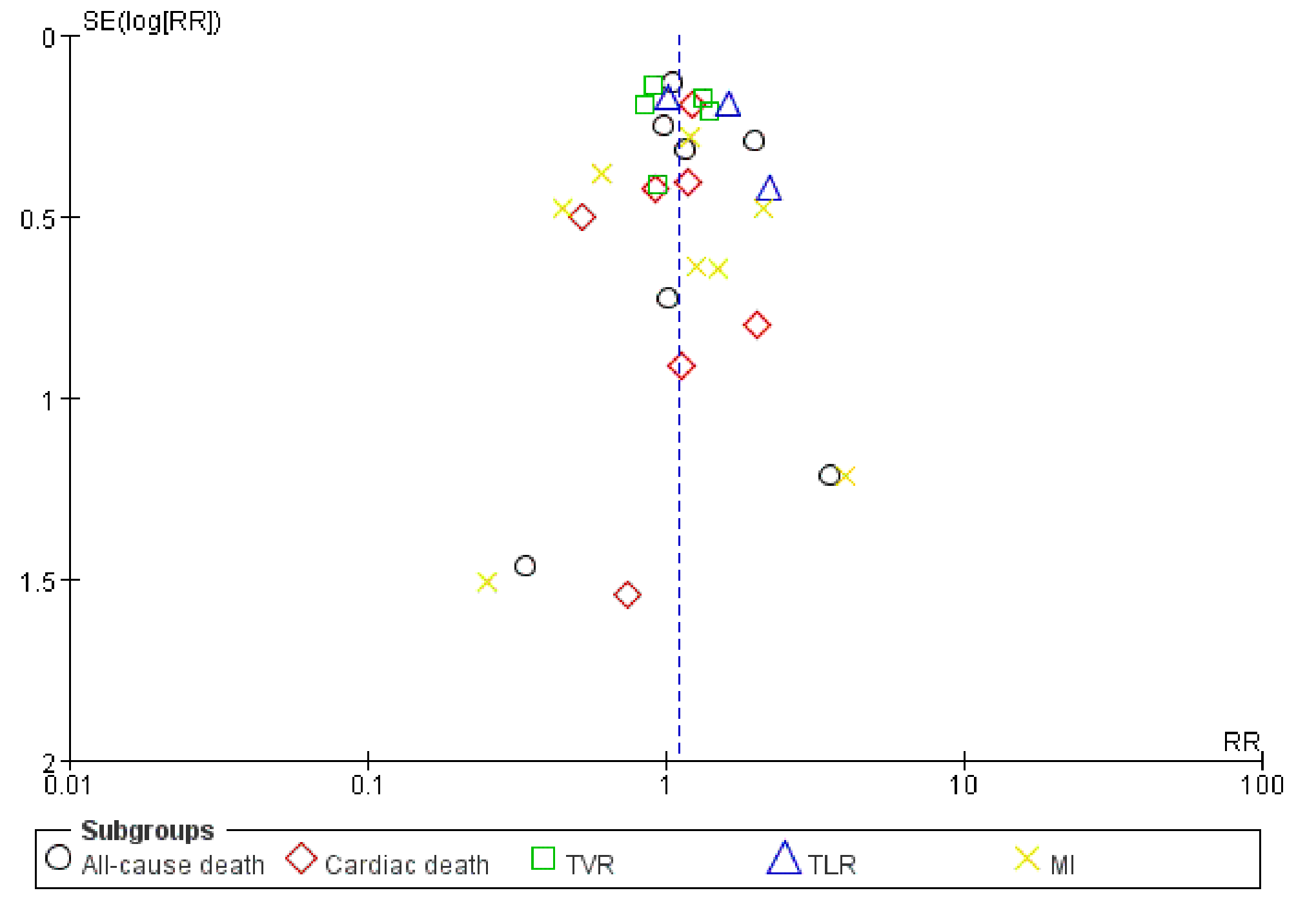

Supplement: Supplementary file 1 — Additional file1: Table S1: Subgroup analysis comparing successful CTO-PCI versus failed CTO-PCI, initial MT in DM patients. Table S2: Subgroup analysis comparing successful CTO-PCI versus failed CTO-PCI, initial MT in non-DM patients. Figure S1: Forest plot comparing MACEs following successful CTO-PCI in patients with versus without DM. MACEs, major adverse cardiac events; CTO, chronic total occlusions; PCI, percutaneous coronary intervention; DM, diabetes mellitus. Figure S2: Funnel plot assessing the publica bias of the studies reporting risk of MACEs following successful CTO-PCI in patients with or without DM. MACEs, major adverse cardiac events; CTO, chronic total occlusions; PCI, percutaneous coronary intervention; DM, diabetes mellitus. Figure S3 Funnel plot assessing the publica bias of the studies reporting risk of MACEs following successful CTO-PCI in patients with or without DM in the subgroups. MACEs, major adverse cardiac events; CTO, chronic total occlusions; PCI, percutaneous coronary intervention; DM, diabetes mellitus. Figure S4 Funnel plot assessing the publica bias of studies reporting the risk of secondary endpoints following successful CTO-PCI in patients with versus without DM. MACEs, major adverse cardiac events; CTO, chronic total occlusions; PCI, percutaneous coronary intervention; DM, diabetes mellitus. [file 12933_2021_1223_MOESM1_ESM.docx]
